# The Utility of Serum Vasorin Levels as a Novel Potential Biomarker for Early Detection of Colon Cancer

**DOI:** 10.7759/cureus.21653

**Published:** 2022-01-26

**Authors:** Mehmet Aydin, Remzi Kızıltan, Sermin Algul, Ozgur Kemik

**Affiliations:** 1 General Surgery, Medical Park Bahçelievler Hospital, Istanbul, TUR; 2 General Surgery, Van Yuzuncu Yil University School of Medicine, Van, TUR; 3 Physiology, Van Yuzuncu Yil University School of Medicine, Van, TUR; 4 Surgical Oncology, Van Yuzuncu Yil University School of Medicine, Van, TUR

**Keywords:** prognosis, vasn, vasorin, biomarker, colonoscopy, crc, colon cancer

## Abstract

Objective

The objective of this study was to investigate the utility of vasorin, a newly discovered transmembrane protein, as a novel biomarker in the early detection of colon cancer.

Methods

A total of 80 patients aged 55-70 years, diagnosed with colon cancer and followed up in our clinics, and 50 healthy volunteer blood donors were included in the study. Participants' demographics such as age, gender, and vasorin levels were recorded and compared between the patient and control groups. In addition, primary tumor status (pT) values N and T stages of the tumors were studied in the patient group. All patients included in the study were pathologically confirmed by colonoscopy plus biopsy and postoperative histopathologic examination.

Results

The mean age was found as 64.59±3.70 (min-max: 55-70) years old in the patient group and 63.56±3.07 (min-max: 57-70) years. There was no statistically significant difference between both groups regarding demographics (p>0.05). Serum Vasorin levels were higher in patients with colon cancer than in the control group (p<0.001). Serum Vasorin levels were higher among patients with advanced disease and related to the clinical stage of the locally advanced tumor.

Conclusion

Our findings revealed that serum vasorin levels are upregulated in patients with colon cancer. Raised vasorin levels may be a non-invasive biomarker beneficial for early detection and prediction of colon cancer prognosis. In addition, vasorin levels further rose as the disease advanced to higher TNM (tumor (T), nodes (N), and metastases (M)) stages. Further comprehensive studies are needed to draw more evident conclusions and generalize our results.

## Introduction

Colorectal cancer (CRC) is the third most frequent malignancy worldwide, with an estimated 1.93 million newly diagnosed CRC cases and 0.94 million CRC-related deaths in 2020 [1]. In addition, CRC is the second leading cause of cancer death globally due to unmet screening programs and therapeutic strategies and increasing incidence. The global number of new CRC cases will reach 3.2 million by 2040 [2]. A broad spectrum of causes play a role in the development of CRC, including modifiable factors such as obesity, smoking, alcohol abuse, diet, sedentary lifestyle and access to health care services, and nonmodifiable factors such as age, gender, family history, and genetics) [3]. It has been reported that at least half of CRC cases could be prevented by reducing modifiable factors, including those related to lifestyle [4]. The increasing incidence of CTC has been associated with the shifting lifestyle and diet towards westernization [5].

Colonoscopy remains the gold standard as a screening tool for tumor detection because it allows the removal of premalignant lesions and enables pathological diagnosis [6]. On the other hand, increasing prevalence of proximal colon cancer, aggressive behavior due to tumor biology and failure to detect a tumor in colonoscopy, and late diagnosis contribute to an increase in mortality rates from CRC [7]. Furthermore, the ongoing devastating COVID-19 pandemic decreased public interest in colonoscopy screening. The COVID-19 pandemic has decreased the number of referred, diagnosed, and treated for CRC [8]. Moreover, the invasive nature of colonoscopy causes patients to often report discomfort or pain during the procedure, which may affect compliance with screening. Although rare, endoscopy also risks developing complications such as perforation and bleeding [9]. This prompted researchers to seek novel, minimally invasive, easy to perform methods for prevention delays in diagnosis and to overcome failure with colonoscopy. Improvements in the sensitivity of the novel stool-based fecal immunochemical test (FIT) and fecal DNA tests, computed tomographic colonography (CTC), and barium enema have attracted interest from researchers. Advancement of screening and diagnostic capacity in CRC requires identifying novel, disease-specific biomarkers. Unfortunately, screening of CRC tissue samples with gene expression assays has failed to identify CRC-related biomarkers with sufficient sensitivity and specificity to be clinically helpful for screening and diagnostic purposes [10]. So far, numerous biomarkers have been studied for early detection of CRC, including APC, MMR, K-ras, p53, Kinases, Bcl-2 Gene, crypt foci, L-DNA, β-Catenin, Cyclin D1, Cyclin E1, and SMAD4. However, the specificity and sensitivity of these biomarkers in the early detection of CRC are limited [11].

Vasorin (VASN), also known as Slit-Like2, is a classic type I transmembrane protein. VASN is abundantly secreted in vascular smooth muscle cells of the aorta and upregulated in breast and hepatocellular cancers, representing a link in tumor progression [12]. VASN has been reported to promote proliferative ability in prostate cancer [13]. Li et al. reported that targeting VASN through small molecular nucleotides is a promising biological therapy for treating hepatocellular carcinoma [12]. To our knowledge, no study in the literature has investigated the link between vasorin and CRC. Therefore, the objective of this study was to investigate the utility of vasorin as a novel biomarker in the early detection of colon cancer.

## Materials and methods

Patients

The local ethics committee approved this prospective study of our hospital (2021/07-08), and the study was carried out per the ethical principles of the Declaration of Helsinki. All participants were informed about the study's objectives in detail and gave informed written consent. 

The study sample was calculated using G-power analysis with a confidence interval of 95%, 5% level of significance, and margin of error of 20%. The study sample was calculated as 75%, and considering loss during the study, we included 80 patients. A total of 80 patients aged 55-70 years, diagnosed with colon cancer and followed up in our clinics, and 50 healthy volunteer blood donors were included in the study. As the study was conducted voluntarily, patients who rejected participation were excluded. Participants' demographics such as age, gender, and vasorin levels were recorded and compared between the patient and control groups. In addition, pT values N and T stages of the tumors were studied in the patient group.

Samples were collected preoperatively from the patients diagnosed with colon cancer (n=80) and blood donors with no evidence of colon cancer (n=50). The patient group consisted of inpatients diagnosed with colon cancer who were admitted to the Yüzüncü Yıl University, Department of General Surgery between May 2019 and May 2021. All patients included in the study were pathologically confirmed by colonoscopy plus biopsy and postoperative histopathologic examination.

Assays

Blood samples of the study groups were stored at -80oC until analysis. Vasorin concentrations were measured by Quantitative Sandwich ELISA (Enzyme-linked immunosorbent assay) kits (Catalog No: MBS085982; MyBioSource; Detection range: 62.5 pg/mL-2000 pg/mL; Sensitivity: 10 pg/mL) according to the manufacturer's instructions. Briefly, 100 µl substrate solution was put into each well, incubated for 20 min at 37oC, and protected from light. 10 µl stop solution was added when gradient color changes were observed in the first 3-4 wells, while not in the last 3-4 wells. Moreover, the reaction was read spectrophotometrically at ~570nm. Most of the samples (93.7%) were taken within three days preoperatively. Samples were centrifuged at 1000g for approximately 20 minutes. The supernatants collected were immediately stored at -80oC until analysis.

Statistical Analysis

Statistical analysis of the data obtained in this study was performed with the NCSS 10 (2015. Kaysville, Utah, USA) software program. The normality of the variables was checked with the Shapiro Wilk and single-sample Kolmogorov Smirnov tests, histogram, Q-Q plot, and box plot graphs and re-expressed as standard deviation, median, minimum, maximum. At the same time, categorical data are given as frequency and percentage. Normally distributed variables were compared using the independent sample t-test and non-normally distributed variables with the Mann-Whitney U test. Groups with three or more categories were compared with Kruskal Wallis's one-way analysis of variance. Multiple comparisons were made with Dunn's test. Nominal variables were evaluated using Chi-square with Yates correction test. P <0.05 values were considered statistically significant.

## Results

**Figure 1:**
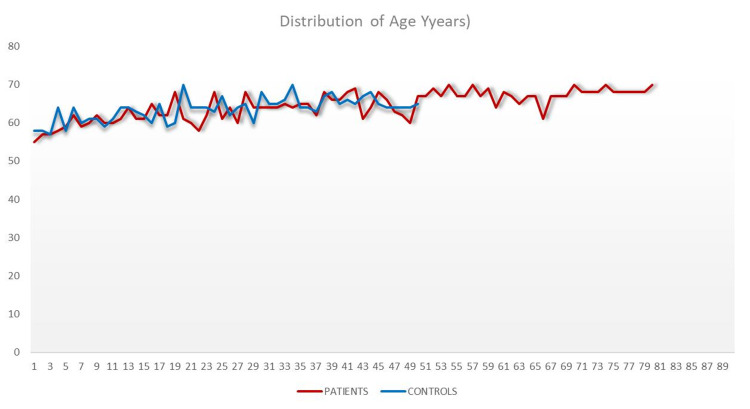
Distribution of patients and control groups in terms of age

A total of 130 patients were included in the study, with 80 in the patient group and 50 in the control group. The mean age was found as 64.59±3.70 (min-max: 55-70) years old in the patient group and 63.56±3.07 (min-max: 57-70) years. In the patient group, the rate of female patients was 44%, while this rate was found as 45% in the control group. No statistically significant difference was found between both groups regarding age (Figure 1). There was no statistically significant difference between both groups in terms of gender (p>0.05).

**Figure 2:**
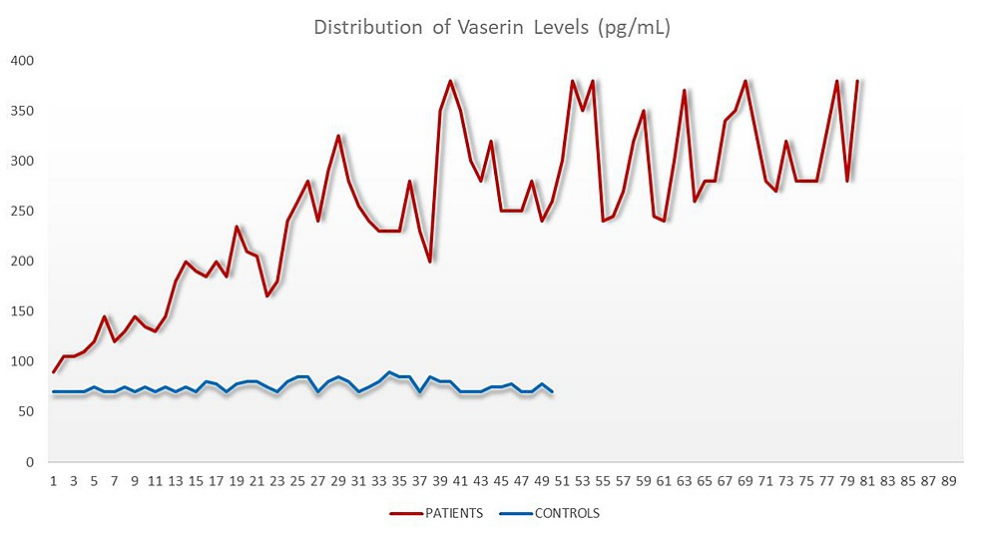
Demographic features and vasorin levels of the groups

Serum Vasorin levels were higher in patients with colon cancer than in the control group (p<0.001, Table 1 and Figure 2).

**Table 1:** Demographic features and vasorin levels of the groups F- female; M- male, pg/mL- picogram/ml

Factor	Control group (n=50)	Patient group (n=80)	p
Age (years)	63.56 ± 3.071	64.58 ± 3.69	>0.05
Gender (F/M)	22/28 44% / 56%	36/44 45% / 55%	
Vasorin (pg/mL)	75.54 ± 5.70	252.81 ± 76.82	<0.001

**Figure 3:**
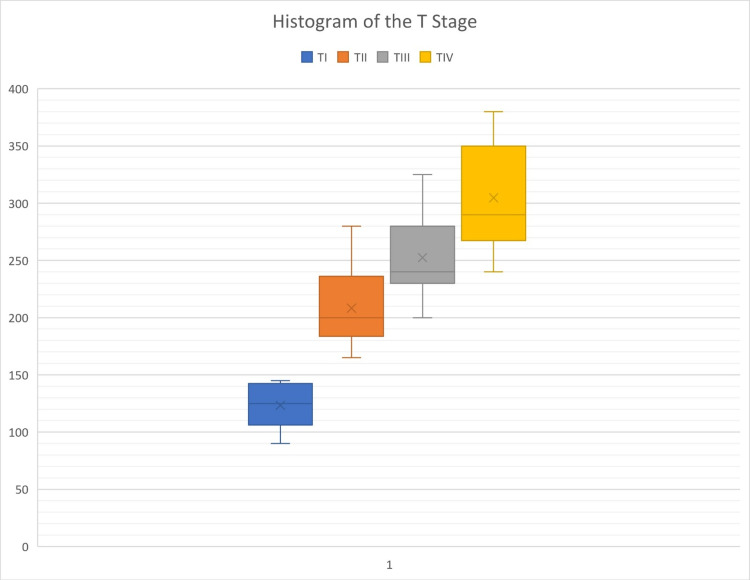
The histogram of the T stage (TI-TIV) T- Tumor status.

When primary tumor status (pT) was evaluated in the patient group; pT1 was found in 8 (10%), pT2 in 8 (10%), pT3 in 22 (27.5%) and pT4 in 42 (52.5%) patients. Based on the TNM (tumor (T), nodes (N), and metastases (M)) staging, TI was found in 12 (15% ), TII in 14 ( 17.5%), TIII in 12 (15% ), and TIV in 42 (52.5%) patients. Nearby spread status was found as N0 in 8 (10%), N1 in 30 (37.5%), and N2 in 42 (52.5%) patients. Metastasis status was found as M0 in 12 (15%) and M1 in 68 (85%) patients. Serum Vasorin levels were higher among patients with advanced disease and related to the clinical stage of locally advanced tumors (Tables 2, 3, and Figure 3).

**Table 2:** Vasorin levels according to the clinical stages of the patients M- Metastasis status; pT- primary tumor status; T- Tumor status.

Factor	Vasorin (pg/mL)				p
	Mean	±SD	Min	Max	
<3cm (n=12)	123.33	18.13	90	145	
>3cm (n=68)	275.66	58.07	165	380	<0.001
M0 (n=12)	123.33	18.13	90	145	
M1 (n=68)	275.66	58.07	165	380	
pT1 (n=8)	115.63	16.99	90	145	
pT2 (n=8)	163.75	27.74	130	200	
pT3 (n=22)	235.91	39.51	165	325	
pT4 (n=42)	304.76	47.47	240	380	<0.001
N0 (n=8)	115.63	16.99	90	145	
N1 (n=30)	216.67	48.6	130	325	
N2 (n=42)	304.76	47.47	240	380	<0.001
TI (n=12)	123.33	18.13	90	145	
TII (n=14)	208.21	33.49	165	280	
TIII (n=12)	252.5	34.73	200	325	
TIV (n=42)	304.76	47.47	240	380	<0.001

**Table 3:** Kruskal-Wallis Multiple-Comparison Z-Value Test (Dunn’s Test) N- Nearby spread status; pT- primary tumor status; T- Tumor status.

Vasorin	pT1	pT2	pT3	pT4
pT1	0.0000	0.7334	2.8704	5.7848
pT2	0.7334	0.0000	1.9823	4.8343
pT3	2.8704	1.9823	0.0000	3.9762
pT4	5.7848	4.8343	3.9762	0.0000
Vasorin	N0	N1	N2	
N0	0.0000	2.4298	5.7848	
N1	2.4298	0.0000	5.2906	
N2	5.7848	5.2906	5.2906	
Vasorin	TI	TII	TII	TIV
TI	0.0000	1.8798	3.2845	6.6115
TII	1.8798	0.0000	1.5287	4.6163
TIII	3.2845	1.5287	0.0000	2.515
TIV	6.6115	4.6163	2.515	0.0000

Serum Vasorin levels were slightly higher in men (p=0.015). However, no significant correlations were observed between serum Vasorin levels and other clinicopathological variables (p>0.05).

## Discussion

In this study, we investigated the utility of vasorin as a novel biomarker in the early diagnosis of colon cancer. We found significantly higher vasorin levels in patients with CRC compared to the control group. In addition, vasorin levels were higher among patients with advanced disease. No statistically significant difference was found between the groups in terms of age and gender.

The estimates of CRC morbidity significantly increase with older ages. Because almost 90% of newly diagnosed CRC occurs in individuals over 50 years old, older age is considered one of the most important factors influencing the risk of developing CRC [14]. The CRC patients are mostly >50 years old, with 80% of colon cancer being ≥ 60 years old at the diagnosis [15]. In our study, the mean age of CRC patients was found as 64.59±3.70 years, consistently with the literature. On the other hand, steadily changing lifestyles towards westernization has increased CRC incidence among younger people, especially in developed countries.

Although colonoscopy remains the gold standard for diagnosing CRC and is generally safe and well-tolerated, mainly when conscious sedation is performed, about one-third of the patients complain of gastrointestinal symptoms including bloating, diarrhea, nausea, and self-limiting bleeding [16]. In addition, approximately seven times increased risk of bleeding and perforation has been observed following polypectomy [17]. This prompted researchers to seek novel, simple, minimally invasive, and easy to obtain biomarkers for early diagnosis of CRC.

An ideal CRC biomarker should be easily and quantitatively measured, highly sensitive and specific, reliable, and reproducible. In addition, such a biomarker should determine patients who require second-line investigations such as endoscopic methods and radiological imaging studies. These goals can be achieved through a non-invasive and inexpensive method using easily available samples collected from urine, breath, serum, and stool [18,19]. So far, a lot of molecules have been studied for this purpose, including tissue biomarkers (cytokeratins, cadherin 17, telomerase, GPA33, Caudal type homeobox 2 [CDX2], etc.), blood biomarkers (Circulating cell-free DNA [cfDNA], MicroRNA [miRNA], Long noncoding RNA [lncRNA], Insulin-like growth factor binding protein 2 [IGFBP-2], etc.) and stool biomarkers (Stool DNA [sDNA], stool miRNA, Fecal immunochemical test [FIT], etc.) [20]. 

Human vasorin (VASN) is a newly discovered membrane protein involved in regulating oxidative stress, vascular endothelial cells, and other activities [21]. It was described for the first time in 2002. VANS is upregulated in some types of tumor cells, serving as a potential biomarker [22]. VASN has been identified in several high-throughput screens as a potential cancer biomarker [12,23]. In addition, VASN has been reported to be a potential therapeutic target for fibroproliferative disorders [24].

In the present study, we investigated the utility of VASN, a classic type I transmembrane protein, as a novel blood biomarker in the early detection of CRC. According to our findings, the mean vasorin level was found as 252.81 ± 76.82 pg/mL in the colon CA group and 75.54 ± 5.70 pg/mL in the healthy group (p<0.001). VASN has been reported to be upregulated in breast cancer and hepatocellular cancer, representing a link in tumor progression [12,25]. Cui et al. reported that VASN promotes the proliferative ability of prostate cancer and thus, aggravates the progression of the disease [13].

Study limitations

This study has some limitations. First, the study was conducted in a single center with a relatively small number of patients. In addition, we could make a regression analysis to determine a cut-off value for vaserin in predicting CRC. However, this can be a subject of further, more comprehensive studies. Finally, the correlation of vasorin levels with other biomarkers of CRC could be analyzed. The prospective design of the study comprises one of its vital aspects. In addition, we think that studying vasorin levels that are easy to obtain non-invasively will contribute to what is known regarding the early detection of colon cancer.

## Conclusions

Our findings revealed that serum vasorin levels are upregulated in patients with colon cancer. Raised vasorin levels may be a non-invasive biomarker beneficial for early detection and prediction of colon cancer prognosis. To our knowledge, this study is the first in the literature to demonstrate the link between vasorin and CRC. In addition, we found that vasorin levels further rose as the disease advanced to higher TNM stages. On the other hand, further comprehensive studies with a larger series of patients are needed to draw more evident conclusions and generalize our results.
